# The Association Between Etiologies and Mortality in Acute Respiratory Distress Syndrome: A Multicenter Observational Cohort Study

**DOI:** 10.3389/fmed.2021.739596

**Published:** 2021-10-18

**Authors:** Yan Wang, Linlin Zhang, Xiuming Xi, Jian-Xin Zhou, The China Critical Care Sepsis Trial (CCCST) Workgroup, Bin Du

**Affiliations:** ^1^Department of Critical Care Medicine, Beijing Tiantan Hospital, Capital Medical University, Beijing, China; ^2^Department of Critical Care Medicine, Fuxing Hospital, Capital Medical University, Beijing, China

**Keywords:** ARDS, etiology, clinical outcome, hospital mortality, pulmonary and extrapulmonary causes

## Abstract

**Background:** Lung-protective ventilation (LPV) strategies have been beneficial in patients with acute respiratory distress syndrome (ARDS). As a vital part of LPV, positive end-expiratory pressure (PEEP) can enhance oxygenation. However, randomized clinical trials of different PEEP strategies seem to show no advantages in clinical outcomes in patients with ARDS. A potential reason is that diverse etiologies and phenotypes in patients with ARDS may account for different PEEP responses, resulting in variations in mortality. We consider hospital mortality to be associated with a more specific classification of ARDS, such as sepsis induced or not, and pulmonary or extrapulmonary one. Our study aimed to compare clinical outcomes in various patients with ARDS by etiologies using the China Critical Care Sepsis Trial (CCCST) database. This was a retrospective analysis of a prospective cohort of 2,138 patients with ARDS in the CCCST database. According to ARDS induced by sepsis or not and medical history, patients were stratified into different four groups. Differences among groups were assessed in hospital mortality, ventilation-free days, and other clinical features.

**Results:** A total of 2,138 patients with ARDS were identified in the database, including 647 patients with sepsis-induced pulmonary ARDS (30.3%), 396 patients with sepsis-induced extrapulmonary ARDS (18.5%), 536 patients with non-sepsis pulmonary ARDS (25.1%), and 559 patients with non-sepsis extrapulmonary ARDS (26.1%). The pulmonary ARDS group had higher mortality compared with the extrapulmonary group (45.9 vs. 23.0%, *p* < 0.01), longer intensive care unit (ICU) and hospital stays (9 vs. 6 days, *p* < 0.01, 20 vs. 18 days, *p* = 0.01, respectively), and fewer ventilation-free days (5 vs. 9 days) in the presence of sepsis. However, the mortality in ARDS without sepsis was inverted compared with extrapulmonary ARDS (pulmonary 23.5% vs. extrapulmonary 29.2%, *p* = 0.04). After adjusting for the Acute Physiology and Chronic Health Evaluation II and sequential organ failure assessment scores and other clinical features, the sepsis-induced pulmonary condition was still a risk factor for death in patients with ARDS (hazard ratio 0.66, 95% CI, 0.54–0.82, *p* < 0.01) compared with sepsis-induced extrapulmonary ARDS and other subphenotypes.

**Conclusions:** In the presence of sepsis, hospital mortality in pulmonary ARDS is higher compared with extrapulmonary ARDS; however, mortality is inverted in ARDS without sepsis. Sepsis-induced pulmonary ARDS should attract more attention from ICU physicians and be cautiously treated.

**Trial registration:** ChiCTR-ECH-13003934. Registered August 3, 2013, http://www.chictr.org.cn.

## Background

Acute respiratory distress syndrome (ARDS) is characterized by refractory cyanosis, a decline in lung compliance, patchy or symmetrical bilateral infiltrates, with high morbidity in intensive care units (ICUs) (1–3). Although lung-protective ventilation (LPV), prone position, and other rescue therapies are applied (2, 4–7) and some new biomedical researches and genomics explorations have been undertaken (8, 9), mortality in ARDS is still relatively high (10).

Positive end-expiratory pressure (PEEP) is a vital part of LPV strategies. A proper level of PEEP has been proved to be beneficial in the clinical outcomes of patients with ARDS (4). However, several recent randomized clinical trials have shown that compared with conventional PEEP setting strategies (the FiO_2_-PEEP table recommended by ARDS-net), patients with ARDS did not benefit from other PEEP strategies (11, 12). A potential explanation is that underlying etiologies in patients with ARDS manifesting various clinical phenotypes have varying responses to PEEP (13). The standard risk factors for ARDS are pneumonia, aspiration of gastric contents, and sepsis (2), which can be defined as two pathogenic pathways leading to ARDS: a pulmonary form—mainly characterized by a pulmonary (direct) insult to the lung—and an extrapulmonary (indirect) condition, in which an indirect trigger impairs the vascular endothelium, activating a systemic inflammatory response. Both forms of ARDS can lead to alveolar damage as a final histopathologic feature (14). Previous studies have shown that ARDS caused by different etiologies or pathogenic pathways differs on CT scans (15, 16) and in respiratory mechanics (17, 18). Interestingly, clinical studies have demonstrated little difference in mortality among ARDS subphenotypes (19, 20). We hypothesized that hospital mortality is associated with specific classifications of ARDS.

Our research, therefore, aimed to compare the hospital mortality of categorized patients with ARDS according to different pathogenic pathways using the China Critical Care Sepsis Trial (CCCST) database (21) to describe clinical characteristics of various etiologies of ARDS (sepsis-induced or not, pulmonary or extrapulmonary). Pulmonary ARDS is mainly caused by pneumonia or trauma without other accompanying infections. Extrapulmonary ARDS is classified as caused by intraabdominal or catheter-related bloodstream infection or central nervous system infection without apparent pneumonia or pulmonary infection symptoms.

## Methods

### Study Population

**Figure 1:**
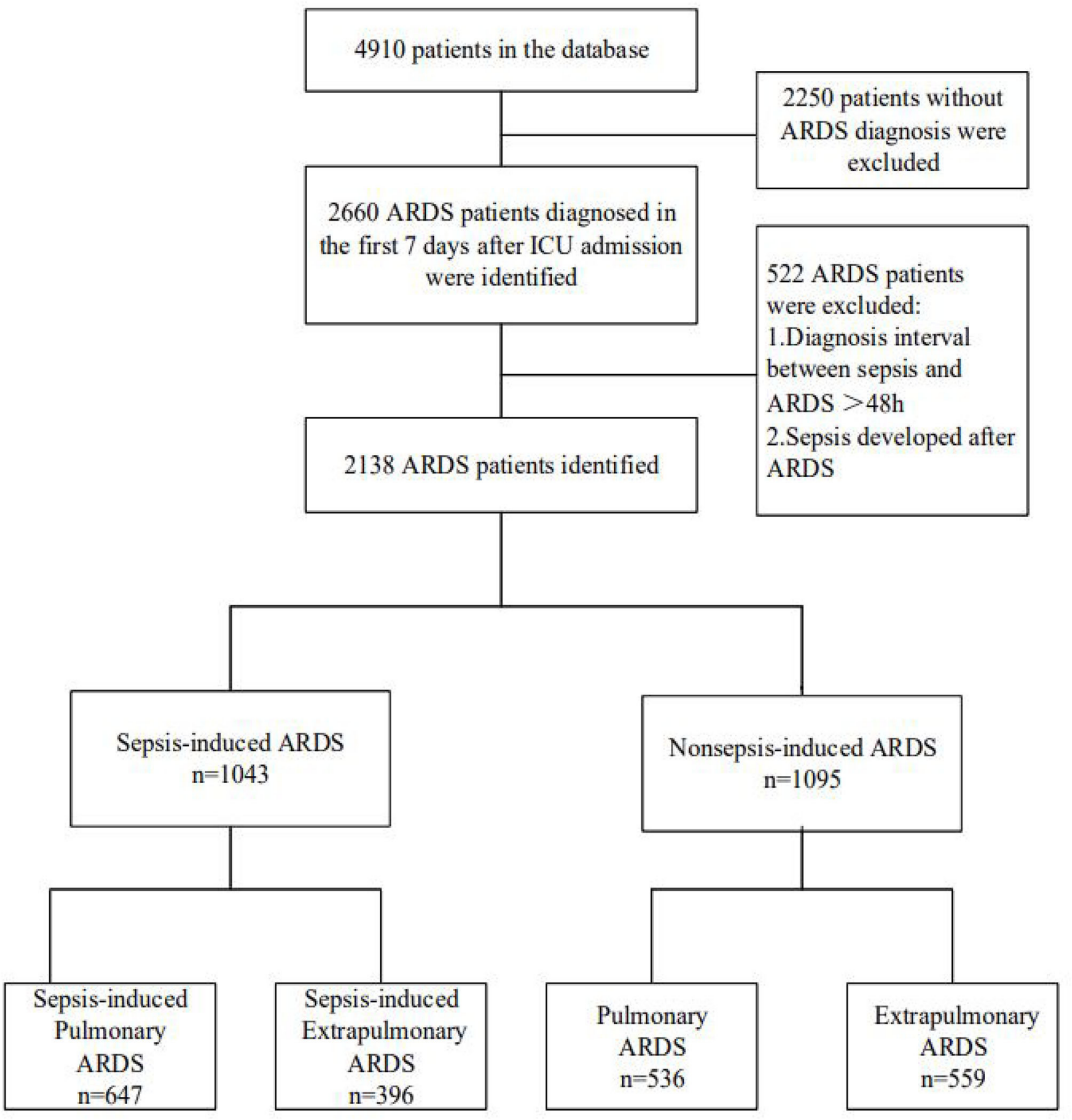
Flowchart of this study.

Data for our research were extracted from the CCCST, which is a prospective, multicenter, observational cohort database including 18 ICUs across China, from January 1, 2014 to August 31, 2015. The study was supported by the National Science and Technology Supporting Plan of the Ministry of Science and Technology of People's Republic of China 2012BAI11B05 (Trial registration ChiCTR, ChiCTRECH13003934; registered August 3, 2013, http://www.chictr.org.cn/proj = 5,633). Data were extracted for adult patients (age ≥18 years) who were admitted consecutively and stayed in ICU for more than 72 h. The primary diagnosis at ICU admission and other comorbidities and complications were recorded every day in the initial 7 days after ICU admission, and clinical outcomes were recorded. ARDS was screened and recorded every day during the first 7 days after ICU admission if the chest X-ray or CT scan of the patient demonstrated new exudate accumulation in the interstitial spaces and lung atelectasis with ventilation support PEEP/continuous positive airway pressure (CPAP) ≥ 5 cm H_2_O with PaO_2_/FiO_2_ <300 mmHg (3). Patients were enrolled if they met these criteria. Sepsis was described as a dedicated infection focus and a quick sequential organ failure assessment (SOFA) score ≥2 within 48 h of admission (22). Sepsis-induced ARDS was regarded as after the time sequence of sepsis and associated with it in 48 h, and ARDS had begun at least 48 h previously (23, 24). If the interval between sepsis of a patient and ARDS diagnosis was more than 48 h or sepsis developed after ARDS in the first 7 days of ICU admission, the patient was excluded (as shown in Figure 1). All patients were stratified into four groups (sepsis-induced pulmonary ARDS, sepsis-induced extrapulmonary ARDS, non-sepsis pulmonary ARDS, and non-sepsis extrapulmonary ARDS), based on the time sequence of development of sepsis and ARDS.

### Data Collection

The baseline data of demographic characteristics, comorbidities, and complications among groups are shown in Table 1. Clinical laboratory values on the day of ARDS diagnosis were used to calculate illness severity scores, for example, Acute Physiology and Chronic Health Evaluation II (APACHE II) (25) and the SOFA scores (26). Less than 10% of the clinical laboratory data used to calculate the illness severity scores were missing in the cohort, missing data were assumed to be of average values and assigned a subscore of 0 (24). Data associated with renal replacement therapy (RRT) and mechanical ventilation (MV) were continuously recorded in the database for 7 days or until discharge from ICU, whichever occurred earlier. Data of RRT and MV applications were extracted from the database. The patient status (survival or death) when discharged from ICU and hospital was extracted. The primary clinical outcome was hospital mortality in different etiologies of ARDS. The secondary clinical outcomes were mechanical ventilation-free days and length of stay in ICU.

**Table 1:** Baseline characteristics of ARDS patients among groups.

	**Sepsis-induced pulmonary ARDS *n* = 647**	**Sepsis-induced extrapulmonary ARDS *n* = 396**	***p***	**Pulmonary ARDS *n* = 536**	**Extrapulmonary ARDS *n* = 559**	***p***
Gender, male, *n* (%)	431 (66.6%)	306 (66.7%)	0.65	339 (63.2%)	377 (67.4%)	0.15
Age, year, median (IQR)	65 (50–78)	63 (51–75)	<0.01	65 (53–77)	58 (45–68)	<0.01
BMI, kg/m^2^ median (IQR)	22.2 (20.2–24.2)	22.7 (20.3–24.9)	<0.01	22.7 (20.2–24.9)	22.5 (20.3–24.5)	0.77
APACHE II score, median (IQR)	21 (16-27)	17 (12-24)	<0.01	13 (8-19)	13 (8-20)	0.51
SOFA median (IQR)	7 (4-10)	6 (2-10)	0.01	2 (2-5)	2 (1-5)	0.96
Admission to ARDS onset, days median (IQR)	1 (1,2)	1 (1-4)	<0.01	3 (1-4)	3 (1-4)	0.07
PaO_2_/FiO_2_, mmHg, median (IQR)	150 (124–198)	151 (142–214)	0.01	150 (150–200)	150 (153–206)	0.11
**Severity**						
Mild, *n* (%)	148 (22.9%)	111 (28.0%)	<0.01	118 (22.0%)	133 (22.8%)	0.53
Moderate, *n* (%)	389 (60.1%)	243 (65.9%)	0.11	333 (62.1%)	335 (59.9%)	0.23
Severe, *n* (%)	110 (17.0%)	42 (10.6%)	0.26	85 (15.9%)	91 (16.3%)	0.70
**Comorbidities and complications**						
Hypertension, *n* (%)	143 (22.1%)	114 (28.8%)	<0.01	182 (34.0%)	178 (31.8%)	0.50
Diabetes mellitus, *n* (%)	82 (12.7%)	79 (19.9%)	0.32	119 (22.2%)	135 (23.2%)	0.49
Liver cirrhosis, *n* (%)	13 (2.0%)	9 (2.3%)	0.86	23 (4.3%)	14 (2.5%)	0.14
Chronic kidney disease, *n* (%)	67 (10.4%)	22 (5.6%)	<0.01	25(4.7%)	35 (6.3%)	0.30
Malignancy, *n* (%)	63 (9.7%)	38 (9.6%)	0.52	86 (16.0%)	35 (5.9%)	<0.01
Acute kidney injury, *n* (%)	235 (36.3%)	242 (61.1%)	0.86	279 (52.1%)	322 (57.6%)	0.07
Acute kidney injury, I	92 (14.2%)	90 (22.7%)	0.17	103(19.2%)	138 (24.7%)	0.04
Acute kidney injury, II	54 (8.3%)	71 (17.9%)	0.30	80 (14.9%)	84 (15.0%)	1.00
Acute kidney injury, III	89 (13.8%)	81 (20.5%)	0.91	96 (17.9%)	100 (17.9%)	1.00
RRT, *n* (%)	157 (24.3%)	70 (17.7%)	0.12	89 (16.6%)	151 (27.0%)	<0.01
**Outcomes**						
No. organ failure, median (IQR)	1(1-1)	1(1-1)	0.01	1(1-1)	1(1-1)	0.03
Ventilation-free days within 28 days, days median (IQR)	5 (5-13)	9 (5-18)	<0.01	13 (11-26)	12 (10-25)	0.06
Length of ICU stay, days median (IQR)	9 (4-20)	6 (3-12)	<0.01	4 (2-11)	4 (2-12)	0.18
Length of hospital stay, days median (IQR)	20 (11-31)	18 (10-29)	0.01	17 (11-27)	17 (19-28)	0.25
ICU mortality, *n* (%)	297 (45.9%)	91 (23.0%)	<0.01	126 (23.5%)	163 (29.2%)	0.04
Hospital mortality, *n* (%)	339 (52.4%)	118(29.8%)	<0.01	181 (33.8%)	199 (35.6%)	0.53

*ARDS, acute respiratory distress syndrome; BMI, body mass index; APACHE II, acute physiology and chronic health evaluation II score; IQR, interquartile range; RRT, renal replacement therapy*.

### Statistical Analysis

Categorical variables are presented as numbers with percentages, and continuous variables are presented as mean ± SD or median with interquartile range. The baseline characteristics of patients were compared using the χ^2^ test for categorical variables and the *t*-test, one-way ANOVA, or Wilcoxon rank-sum test for continuous variables, as appropriate. The Kaplan–Meier method was used to compare hospital mortality and survival probability among groups. A multiple proportional hazards model was established to detect risk factors for hospital mortality. The log-rank test and proportional hazards assumption was conducted to assess the model. Significance was set at a two-sided *p* < 0.05. Analyses were performed with R software (version 4.03 http://www.r-project.org). The G-power software (version 3.1 Franz Faul, University Kiel, Germany) (27) was applied to estimate the sample size and statistical power.

## Results

**Figure 2:**
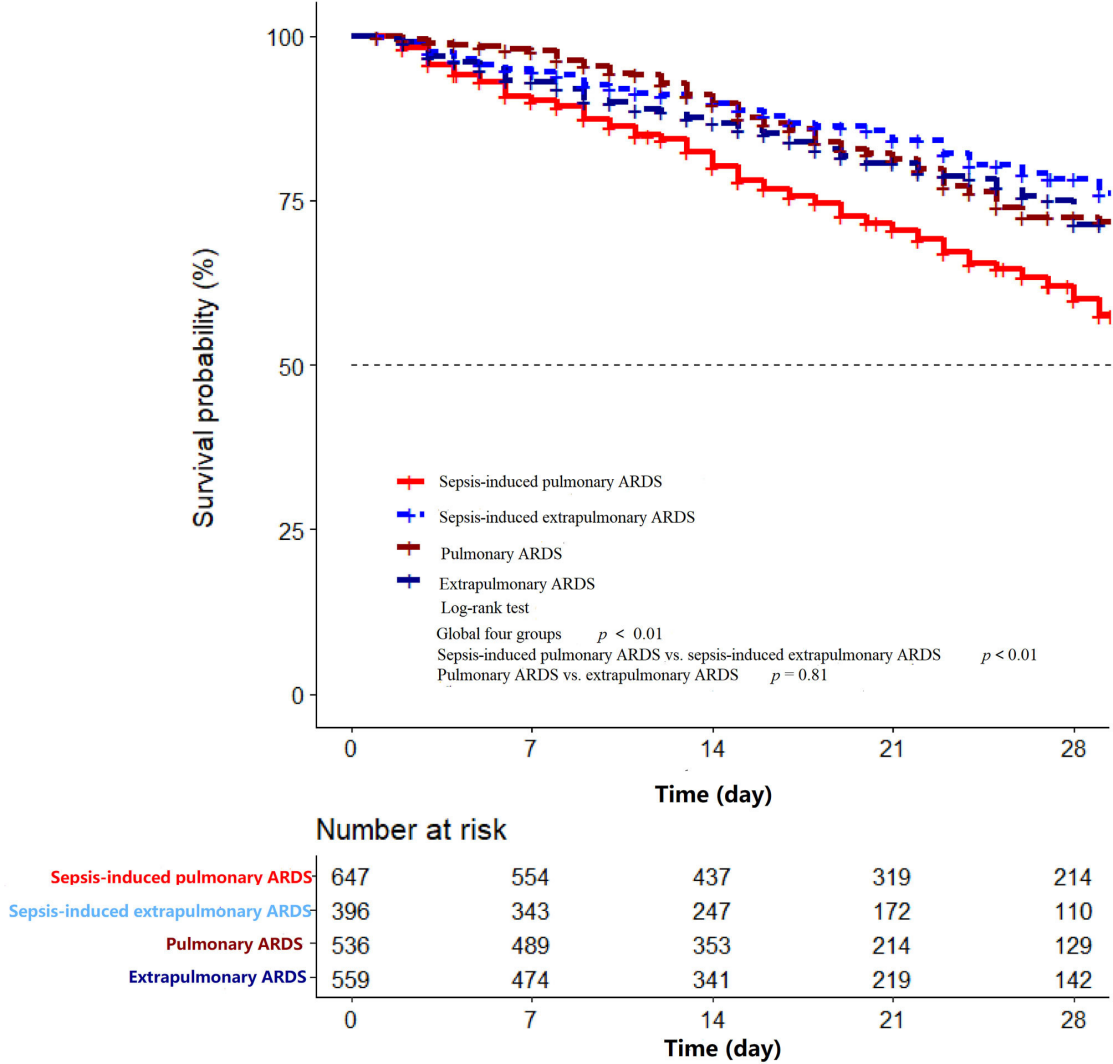
The Kaplan–Meier curve of hospital mortality among sepsis-induced pulmonary ARDS, sepsis-induced extrapulmonary ARDS, pulmonary ARDS, and extrapulmonary ARDS groups in the first 28 days after ICU admission. In the global log-rank test *p* < 0.01. The log-rank test of sepsis-induced pulmonary ARDS vs. sepsis-induced extrapulmonary ARDS was *p* < 0.01; for pulmonary ARDS vs. extrapulmonary ARDS *p* = 0.81.

A total of 2,138 patients with ARDS were identified in the database, namely, 647 patients with sepsis-induced pulmonary ARDS (30.3%), 396 patients with sepsis-induced extrapulmonary ARDS (18.5%), 536 patients with non-sepsis pulmonary ARDS (25.1%), and 559 patients with non-sepsis extrapulmonary ARDS (26.1%). The ventilation-free days within 28 days in each group were 5, 9, 13, and 12 days, respectively (sepsis-induced pulmonary vs. extrapulmonary ARDS *p* < 0.01, non-sepsis pulmonary vs. extrapulmonary ARDS *p* = 0.06). The duration of ICU stay were 9, 6, 4, and 4 days (sepsis-induced pulmonary vs. extrapulmonary ARDS *p* < 0.01, non-sepsis pulmonary vs. extrapulmonary ARDS *p* = 0.18) and hospital stay were 20, 18, 17, and 17 days in every group separately (sepsis-induced pulmonary vs. extrapulmonary ARDS *p* = 0.01, non-sepsis pulmonary vs. extrapulmonary ARDS *p* = 0.25, detailed in Table 1). According to the Kaplan–Meier method, the sepsis-induced pulmonary ARDS demonstrated as a risk factor of death among patients with ARDS after the log-rank test (in the global log-rank test *p* < 0.01). The log-rank test of sepsis-induced pulmonary ARDS vs. sepsis-induced extrapulmonary ARDS was *p* < 0.01; for pulmonary ARDS vs. extrapulmonary ARDS, *p* = 0.81. The multiple proportional Cox hazards model results indicated that a high APACHE II score (hazard ratio (HR) 1.00, 95% CI: 0.99–1.00, *p* < 0.01), aging (HR 1.01, 95% CI: 0.99–1.01, *p* = 0.02), and male (HR 1.24, 95% CI: 0.07–1.44, *p* = 0.01) were risk factors for death. Among the four clinical phenotypes of ARDS, sepsis-induced pulmonary ARDS remained a risk factor for death after adjusting for other clinical features (as shown in Figure 2). According to our result, the statistical power was 0.93.

## Discussion

To our knowledge, this research is the largest multicenter cohort to examine clinical outcomes in patients with pulmonary and extrapulmonary ARDS. In this retrospective analysis of prospective registry study carried out in 18 ICUs in mainland China, ARDS was still an important complication in ICU, with high hospital mortality. The hospital mortality, on the whole, was 31.67%, similar to that found in previous research (19) (pulmonary vs. extrapulmonary 44 vs. 33.2%).

**Figure 3:**
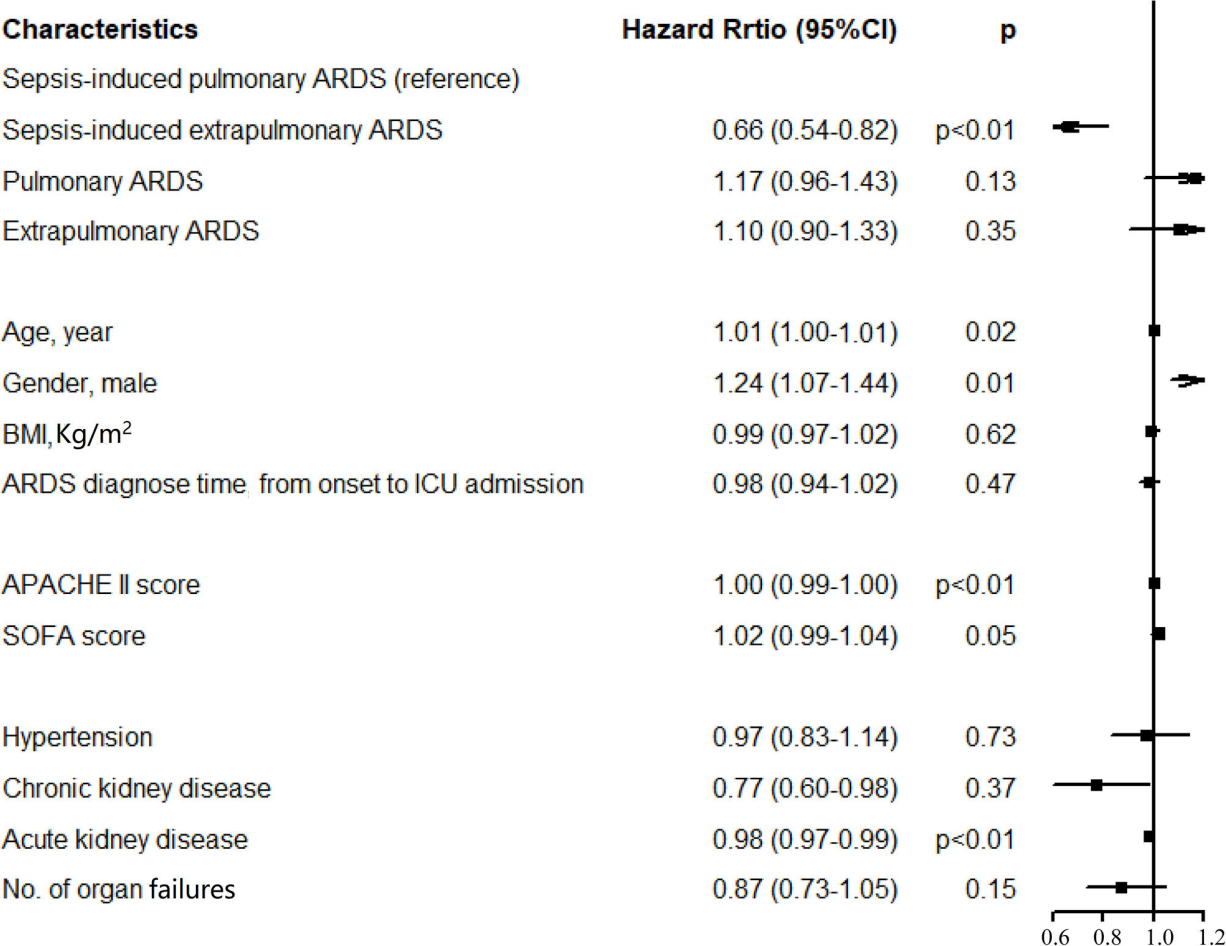
Results of multivariate Cox regression analysis. After the proportional hazard assumption, APACHE II and morbidity with acute kidney injury were transformed into time-dependent covariables.

In the sepsis-induced ARDS group, the pulmonary group of patients had higher APACHE II and SOFA scores, which may account for increased mortality compared with extrapulmonary ARDS. Additionally, the sepsis-induced pulmonary group had higher hospital mortality, extended ICU stay (both *p* < 0.01), and shorter ventilation-free days compared with the extrapulmonary group (9 vs. 5 days, *p* < 0.01). In addition, in the non-sepsis ARDS group, ventilation-free days, length of ICU and hospital stays, and hospital mortality in pulmonary and extrapulmonary groups were comparable, except for the cases of organ failure and ICU mortality. Among the four clinical phenotypes of ARDS, the sepsis-associated pulmonary ARDS demonstrated the highest mortality through using the Kaplan–Meier method (global long-rank test *p* < 0.01, Figure 3). The result of our research reveals that pulmonary ARDS had the highest mortality in the presence of sepsis compared with the other three phenotype groups. In the presence of sepsis, the hospital mortality in pulmonary ARDS is higher compared with extrapulmonary ARDS. However, hospital mortality in pulmonary ARDS without sepsis is lower than extrapulmonary ARDS. The result is partially controversial to our clinical recognition.

Previous cohort studies (19, 20) have shown subtle distinctions are exhibited clinically but that there is no difference in hospital mortality between pulmonary and extrapulmonary ARDS, which is partly inconsistent with our findings. Our research indicated that hospital mortality of ARDS without sepsis is comparable, no matter if caused by pulmonary or extrapulmonary factors (according to our result, mortality in extrapulmonary ARDS without sepsis was slightly higher). In contrast, inversely, sepsis-induced pulmonary ARDS had the highest mortality among the four groups. In the early stage of ARDS, the pulmonary type is always characterized by a worse impairment of gas exchange and a greater potential of lung recruitment compared with extrapulmonary one (16, 28). Secondary analysis research has also implied that patients susceptible to the higher potential of recruitment ARDS exhibited high mortality; however, that cluster of ARDS is always caused by pulmonary factors, accounting for 63.6% of cases (29). Although the respiratory mechanics data were not available in this research, the increased mortality in the sepsis-induced pulmonary ARDS group could also be explained in clinical and preclinical experiments (18, 28).

The subphenotype of patients with ARDS caused by pneumonia-associated sepsis could not be classified solely into pulmonary ARDS because these patients had higher APACHE II and SOFA scores than the extrapulmonary group, and the primary risk factor in ARDS is sepsis. Sepsis, a classic cause of extrapulmonary ARDS, is a multifaceted host response to inflammation (22). In the early stage of the immune response process, inflammatory cytokines and other endogenous factors may be amplified (29), placing patients in an unstable and dangerous situation. Luo et al. (20) classified ARDS developed in pneumonia-associated sepsis as pulmonary and concluded that mortality in patients with direct ARDS was similar to that in patients with indirect ARDS, although this seems unlikely. Sepsis is a classic risk factor of ARDS as an extrapulmonary one (1). However, compared with extrapulmonary ARDS, pulmonary one is always paralleled with a high potential of recruitment (28, 30), demonstrating various responses to PEEP and gas exchanges. According to our findings, ARDS caused by pneumonia-associated sepsis seems to be severer than one associated with either pneumonia or sepsis.

A cohort study (31) indicated that compared with the low potential in the recruitment group, a higher percentage of the recruitable lung group had high mortality (as high as 41%), in which 56% had pulmonary ARDS and 24% had an unrecognized subtype of ARDS. This is partially consistent with our finding that pulmonary ARDS is more severe when sepsis is involved. Some researchers also proposed (28), consistent with our study, that in the early stage of ARDS, recognition of the origins of ARDS is more critical than customized ventilation management. However, we should be cautious about assessing and stratifying patients with ARDS with additional clinical features because the LIVE study (32) revealed that when using lung morphology to inform ventilatory management, as many as 21% of patients with ARDS could be misclassified, which may result in increased mortality rates.

There are several limitations to our research. Data on respiratory mechanics were unavailable to further describe the association between mechanics and clinical outcomes, which is the most significant limitation to our study. Due to the inaccessible data on respiratory mechanics in the established database, further description of the association between mechanics and clinical outcomes was unavailable. This study suggests that other studies on respiratory mechanics according to phenotypes of ARDS are needed. The study was a multicenter, observational cohort study, and hospital mortalities among different regions may be influenced by local medical practices and policies. Moreover, there are no specific guidelines for stratifying sepsis-induced ARDS or pulmonary and extrapulmonary cases. Our findings may also be affected by other underlying clinical characteristics we did not study. Such characteristics may discriminate among various clinical subphenotypes of ARDS and maybe focus on in future research.

## Conclusion

This study is a snapshot of mortality for different etiologies of ARDS, revealing that mortalities in ARDS without sepsis are similar, no matter whether caused by pulmonary or extrapulmonary factors. However, ARDS developed in the presence of sepsis, mainly induced by pulmonary factors, should attract more attention from ICU physicians and be treated with caution.

## Data Availability Statement

The raw data supporting the conclusions of this article will be made available by the authors, without undue reservation.

## Ethics Statement

The study protocol was approved by the Ethics Committees of Fuxing Hospital, Capital Medical University (approval notice number 2013FXHEC-KY018), and all other centers. Written informed consent for participation was not required for this observational survey. The patient records and information were anonymized before analysis.

## Author Contributions

J-XZ and XX conceived of, designed, and supervised the study. YW participated in the data recording and wrote the drafts of the manuscript. LZ finalized the analysis, designed the study, and interpreted the findings. All authors read and approved the final manuscript, authors contributed to the article, and approved the submitted version.

## Funding

This work was supported by the National Science and Technology Supporting Plan of the Ministry of Science and Technology of the People's Republic of China (2012BAI11B05). The funding source had no role in writing the manuscript or the decision to submit it for publication.

## Conflict of Interest

The authors declare that the research was conducted in the absence of any commercial or financial relationships that could be construed as a potential conflict of interest.

## Publisher's Note

All claims expressed in this article are solely those of the authors and do not necessarily represent those of their affiliated organizations, or those of the publisher, the editors and the reviewers. Any product that may be evaluated in this article, or claim that may be made by its manufacturer, is not guaranteed or endorsed by the publisher.

## Members of the CCCST Workgroup

Bin Du, Medical Intensive Care Unit, Peking Union Medical College Hospital, Beijing, China; Li Weng, Medical Intensive Care Unit, Peking Union Medical College Hospital, Beijing, China; Tong Li, Department of Critical Care Medicine, Beijing Tongren Hospital, Capital Medical University, Beijing, China; Meili Duan, Department of Critical Care Medicine, Beijing Friendship Hospital, Capital Medical University, Beijing, China; Wenxiong Li, Surgical Intensive Care Unit, Beijing Chaoyang Hospital, Capital Medical University, Beijing, China; Bing Sun, Department of Respiratory and Critical Care Medicine, Beijing Institute of Respiratory Medicine, Beijing Chaoyang Hospital, Capital Medical University, Beijing, China; Jianxin Zhou, Department of Critical Care Medicine, Beijing Tiantan Hospital, Capital Medical University, Beijing, China; Jianguo Jia, Surgical Intensive Care Unit, Xuanwu Hospital, Capital Medical University, Beijing, China; Xi Zhu, Department of Critical Care Medicine, Peking University Third Hospital, Beijing, China; Qingyuan Zhan, Department of Critical Care Medicine, China-Japan Friendship Hospital, Beijing, China; Xiaochun Ma, Department of Critical Care Medicine, The First Affiliated Hospital of China Medical University, Shenyang, China; Tiehe Qin and Shouhong Wang, Department of Critical Care Medicine, Guangdong Geriatric Institute, Guangdong General Hospital, Guangdong, China; Yuhang Ai, Department of Critical Care Medicine, Xiangya Hospital, Central South University, Changsha, China; Yan Kang and Xuelian Liao, Department of Critical Care Medicine, West China Hospital, Sichuan University, Sichuan, China; Xiangyuan Cao, Department of Critical Care Medicine, General Hospital of Ningxia Medical University, Ningxia, China; Yushan Wang, Intensive Care Unit, The First Hospital of Jilin University, Changchun, China; and Duming Zhu, Surgical Intensive Care Unit, Department of Anaesthesiology, ZhongShan Hospital, FuDan University, Shanghai, China. The CCCST group performed the recruitment and data collection.

## Abbreviations

ARDS: Acute respiratory distress syndrome
LPV: Lung protective ventilation
PEEP: Positive end-expiratory pressure
CPAP: Continuous Positive Airway Pressure
CCCST: The China Critical Care Sepsis Trail
ICU: intensive care units
HR: harazed ratio
APACHE II: Acute Physiology and Chronic Health Evaluation II score
IQR: Interquartile range
RRT: renal replacement therapy
BMI: body mass index.
